# 2-[1-(2-Hy­droxy-6-meth­oxy­phen­yl)ethyl­idene]-*N*-methyl­hydrazinecarbothio­amide acetonitrile monosolvate

**DOI:** 10.1107/S1600536812048799

**Published:** 2012-12-05

**Authors:** Brian J. Anderson, Alexander M. Keeler, Kelly A. O’Rourke, Shannon T. Krauss, Jerry P. Jasinski

**Affiliations:** a Department of Chemistry, Keene State College, 229 Main Street, Keene, NH 03435–2001, USA

## Abstract

In the title compound, C_11_H_15_N_3_O_2_S·C_2_H_3_N, the dihedral angle between the benzene ring and the mean plane of the hydrazinecarbothio­amide group is 75.1 (2)°. In the crystal, the main mol­ecule is linked to the solvent mol­ecule by a weak N—H⋯N hydrogen bond while O—H⋯S hydrogen bonds link the mol­ecules into columns along [100].

## Related literature

For thio­semicarbazone structures and their biological activity, see: Lobana *et al.* (2009 ▶). For thio­semicarbazone as ligands for hydrogenations or metal-catalysed reactions, see: Pelagatti *et al.* (1998 ▶); Xie *et al.* (2010 ▶). For a related structure, see: Anderson *et al.* (2012 ▶). For standard bond lengths, see: Allen *et al.* (1987 ▶).
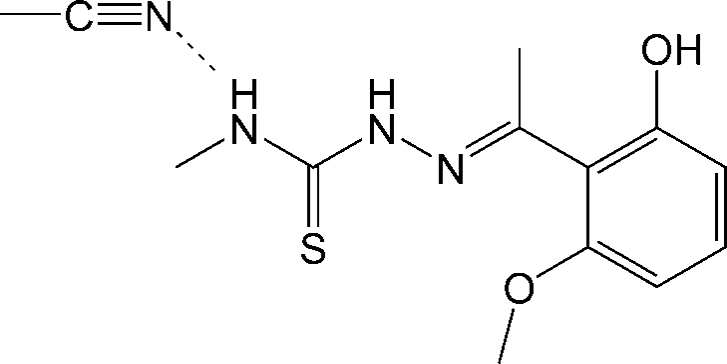


## Experimental

### 

#### Crystal data

C_11_H_15_N_3_O_2_S·C_2_H_3_N
*M* *_r_* = 294.38
Triclinic, 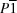

*a* = 7.6232 (10) Å
*b* = 9.4004 (9) Å
*c* = 11.8031 (12) Å
α = 80.121 (8)°
β = 71.555 (10)°
γ = 74.732 (10)°
*V* = 770.44 (16) Å^3^
*Z* = 2
Cu *K*α radiation
μ = 1.93 mm^−1^
*T* = 173 K
0.44 × 0.28 × 0.12 mm

#### Data collection

Agilent Xcalibur (Eos, Gemini CCD) diffractometer
Absorption correction: multi-scan (*CrysAlis RED*; Agilent, 2012 ▶) *T*_min_ = 0.575, *T*_max_ = 1.000
4716 measured reflections
2968 independent reflections
2622 reflections with *I* > 2σ(*I*)
*R*_int_ = 0.020

#### Refinement

*R*[*F* ^2^ > 2σ(*F* ^2^)] = 0.063
*wR*(*F* ^2^) = 0.170
*S* = 1.06
2968 reflections
186 parameters
H-atom parameters constrained
Δρ_max_ = 1.13 e Å^−3^
Δρ_min_ = −0.26 e Å^−3^

### 

Data collection: *CrysAlis PRO* (Agilent, 2012 ▶); cell refinement: *CrysAlis PRO*; data reduction: *CrysAlis RED* (Agilent, 2012 ▶); program(s) used to solve structure: *SHELXS97* (Sheldrick, 2008 ▶); program(s) used to refine structure: *SHELXL97* (Sheldrick, 2008 ▶); molecular graphics: *SHELXTL* (Sheldrick, 2008 ▶); software used to prepare material for publication: *SHELXTL*.

## Supplementary Material

Crystal structure: contains datablock(s) global, I. DOI: 10.1107/S1600536812048799/rk2388sup1.cif

Structure factors: contains datablock(s) I. DOI: 10.1107/S1600536812048799/rk2388Isup2.hkl

Supplementary material file. DOI: 10.1107/S1600536812048799/rk2388Isup3.cml

Additional supplementary materials:  crystallographic information; 3D view; checkCIF report

## Figures and Tables

**Table 1:** Hydrogen-bond geometry (Å, °)

*D*—H⋯*A*	*D*—H	H⋯*A*	*D*⋯*A*	*D*—H⋯*A*
O2—H2⋯S1^i^	0.84	2.34	3.1823 (17)	177
N1—H1⋯N1*A*	0.88	2.25	3.039 (3)	149

Symmetry code: (i) 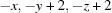.

**Fig. 1.**
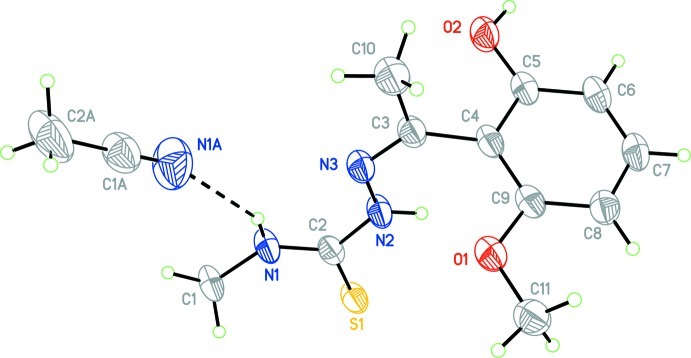
The molecular structure of the title compound with the atom numbering scheme. Displacement ellipsoids are drawn at the 50% probability level. A weak H—H···N intramolecular interaction with the solvent acetonitrile molecule is shown with a dashed line. H atoms are presented as small spheres of arbitrary radius.

**Fig. 2.**
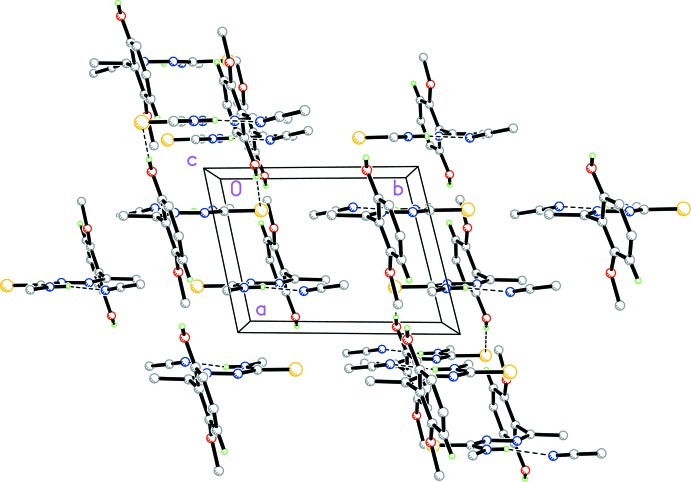
Packing diagram viewed along the c axis. Weak H—H···N intramolecular interactions and O—H···S intermolecular interactions which link the molecules into columns along [1 0 0] are shown by dashed lines.

## supplementary crystallographic information

### Comment

Thiosemicarbazones are an important class of ligands and their metal complexes and biological activity have been investigated (Lobana, *et al.*, 2009). More recently, thiosemicarbazones have been studied as ligands for metal catalyzed reactions such as Mizoroki–Heck couplings (Xie *et al.*, 2010) and hydrogenations (Pelagatti *et al.*, 1998). A similar and related structure has been reported (Anderson, *et al.*, 2012). The crystal structure of a novel thiosemicarbazone molecule, C_13_H_18_N_4_O_2_S, I, is reported here.

In I the dihedral angle between least squares planes of the benzene ring (C4–C9) and hydrazinecarbothioamide (N1/C2/S1/N2/N3) group is 75.1 (2)° (Fig. 1). Bond lengths are in normal ranges (Allen *et al.*, 1987). Weak H—H···N intramolecular interactions and O—H···S intermolecular interactions (Table 1) link the molecules into columns along [1 0 0] (Fig. 2).

### Experimental

A 50 mL round bottom flask was charged with 0.208 g (1.2 mmol) of 2'-hydroxy-6'-methoxyacetophenone and 0.126 g (1.2 mmol) of 4-methyl-3-thiosemicarbazide followed by 20 mL of methanol, resulting in a clear yellow solution. The solution was refluxed for 48 hours, and then the solvent was removed by rotary evaporation. The product was dissolved into acetonitrile at 313 K and slowly allowed to cool to 273 K. Translucent crystals were observed after 48 hours. M.p.: 415–420 K.

### Refinement

All of the H atoms were placed in their calculated positions and then refined using the riding model with C—H lengths of 0.95Å (CH), 0.96Å (CH_3_), 0.88Å (NH) or 0.84Å (OH). The isotropic displacement parameters for these atoms were set to 1.18 to 1.20 (CH), 1.20 (NH, OH) or 1.50 (CH_3_) times *U*_eq_ of the parent atom.

### Crystal data

**Table:**

C_11_H_15_N_3_O_2_S·C_2_H_3_N	*Z* = 2
*M**_r_* = 294.38	*F*(000) = 312
Triclinic, *P*1	*D*_x_ = 1.269 Mg m^−^^3^
Hall symbol: -P 1	Melting point = 415–420 K
*a* = 7.6232 (10) Å	Cu *K*α radiation, λ = 1.54184 Å
*b* = 9.4004 (9) Å	Cell parameters from 2112 reflections
*c* = 11.8031 (12) Å	θ = 4.0–72.3°
α = 80.121 (8)°	µ = 1.93 mm^−^^1^
β = 71.555 (10)°	*T* = 173 K
γ = 74.732 (10)°	Chunk, colourless
*V* = 770.44 (16) Å^3^	0.44 × 0.28 × 0.12 mm

### Data collection

**Table:**

Agilent Xcalibur (Eos, Gemini CCD) diffractometer	2968 independent reflections
Radiation source: Enhance (Cu) X–ray Source	2622 reflections with *I* > 2σ(*I*)
Graphite monochromator	*R*_int_ = 0.020
Detector resolution: 16.0416 pixels mm^-1^	θ_max_ = 72.4°, θ_min_ = 4.0°
ω scans	*h* = −9→9
Absorption correction: multi-scan (*CrysAlis PRO* and *CrysAlis RED*; Agilent, 2012)	*k* = −7→11
*T*_min_ = 0.575, *T*_max_ = 1.000	*l* = −12→14
4716 measured reflections	

### Refinement

**Table:**

Refinement on *F*^2^	Primary atom site location: structure-invariant direct methods
Least-squares matrix: full	Secondary atom site location: difference Fourier map
*R*[*F*^2^ > 2σ(*F*^2^)] = 0.063	Hydrogen site location: inferred from neighbouring sites
*wR*(*F*^2^) = 0.170	H-atom parameters constrained
*S* = 1.06	*w* = 1/[σ^2^(*F*_o_^2^) + (0.115*P*)^2^ + 0.1027*P*] where *P* = (*F*_o_^2^ + 2*F*_c_^2^)/3
2968 reflections	(Δ/σ)_max_ < 0.001
186 parameters	Δρ_max_ = 1.13 e Å^−^^3^
0 restraints	Δρ_min_ = −0.26 e Å^−^^3^

### Special details

**Table:**

Geometry. All s.u.'s (except the s.u. in the dihedral angle between two l.s. planes) are estimated using the full covariance matrix. The cell s.u.'s are taken into account individually in the estimation of s.u.'s in distances, angles and torsion angles; correlations between s.u.'s in cell parameters are only used when they are defined by crystal symmetry. An approximate (isotropic) treatment of cell s.u.'s is used for estimating s.u.'s involving l.s. planes.
Refinement. Refinement of *F*^2^ against ALL reflections. The weighted *R*–factor *wR* and goodness of fit *S* are based on *F*^2^, conventional *R*–factors *R* are based on *F*, with *F* set to zero for negative *F*^2^. The threshold expression of *F*^2^ > σ(*F*^2^) is used only for calculating *R*–factors(gt) *etc*. and is not relevant to the choice of reflections for refinement. *R*–factors based on *F*^2^ are statistically about twice as large as those based on *F*, and *R*–factors based on ALL data will be even larger.

### Fractional atomic coordinates and isotropic or equivalent isotropic displacement parameters (Å^2^)

**Table:**

	*x*	*y*	*z*	*U*_iso_*/*U*_eq_	
S1	0.25112 (8)	1.21212 (6)	0.65436 (4)	0.0389 (2)	
O1	0.6434 (2)	0.76106 (19)	0.83823 (13)	0.0433 (4)	
O2	0.0253 (2)	0.7583 (2)	1.08053 (14)	0.0453 (4)	
H2	−0.0446	0.7645	1.1514	0.068*	
N1	0.2464 (3)	0.9666 (2)	0.57065 (15)	0.0378 (4)	
H1	0.2499	0.8710	0.5813	0.045*	
N2	0.2694 (3)	0.94030 (19)	0.76141 (15)	0.0362 (4)	
H2A	0.2792	0.9762	0.8227	0.043*	
N3	0.2683 (2)	0.79307 (19)	0.76798 (15)	0.0345 (4)	
C1	0.2309 (3)	1.0463 (3)	0.45605 (19)	0.0430 (5)	
H1A	0.2248	0.9777	0.4042	0.064*	
H1B	0.3419	1.0893	0.4176	0.064*	
H1C	0.1155	1.1255	0.4691	0.064*	
C2	0.2555 (3)	1.0302 (2)	0.66005 (18)	0.0330 (4)	
C3	0.2951 (3)	0.7115 (2)	0.86217 (18)	0.0342 (5)	
C4	0.3366 (3)	0.7630 (2)	0.96197 (17)	0.0334 (5)	
C5	0.1988 (3)	0.7793 (2)	1.07313 (18)	0.0360 (5)	
C6	0.2433 (3)	0.8147 (2)	1.16953 (19)	0.0403 (5)	
H6	0.1500	0.8267	1.2449	0.048*	
C7	0.4234 (3)	0.8320 (2)	1.15433 (19)	0.0404 (5)	
H7	0.4531	0.8546	1.2206	0.049*	
C8	0.5626 (3)	0.8174 (2)	1.0454 (2)	0.0378 (5)	
H8	0.6857	0.8310	1.0363	0.045*	
C9	0.5175 (3)	0.7824 (2)	0.94952 (18)	0.0348 (5)	
C10	0.2904 (4)	0.5518 (2)	0.8712 (2)	0.0459 (6)	
H10A	0.2653	0.5322	0.7996	0.069*	
H10B	0.1898	0.5289	0.9426	0.069*	
H10C	0.4129	0.4896	0.8774	0.069*	
C11	0.8359 (3)	0.7636 (3)	0.8237 (2)	0.0465 (6)	
H11A	0.9138	0.7373	0.7433	0.070*	
H11B	0.8831	0.6921	0.8841	0.070*	
H11C	0.8428	0.8632	0.8337	0.070*	
N1A	0.2277 (5)	0.6691 (3)	0.5126 (3)	0.0736 (8)	
C1A	0.2497 (4)	0.5798 (3)	0.4541 (2)	0.0530 (6)	
C2A	0.2725 (7)	0.4659 (4)	0.3788 (3)	0.0844 (12)	
H2AA	0.2316	0.5115	0.3076	0.127*	
H2AB	0.1952	0.3951	0.4238	0.127*	
H2AC	0.4062	0.4141	0.3541	0.127*	

### Atomic displacement parameters (Å^2^)

**Table:**

	*U*^11^	*U*^22^	*U*^33^	*U*^12^	*U*^13^	*U*^23^
S1	0.0540 (4)	0.0364 (3)	0.0265 (3)	−0.0125 (2)	−0.0111 (2)	−0.0006 (2)
O1	0.0434 (8)	0.0578 (10)	0.0286 (8)	−0.0144 (7)	−0.0074 (6)	−0.0046 (7)
O2	0.0443 (9)	0.0651 (11)	0.0280 (8)	−0.0151 (7)	−0.0071 (6)	−0.0096 (7)
N1	0.0521 (11)	0.0381 (9)	0.0228 (8)	−0.0071 (8)	−0.0123 (7)	−0.0041 (7)
N2	0.0521 (10)	0.0347 (9)	0.0246 (8)	−0.0098 (7)	−0.0147 (7)	−0.0029 (7)
N3	0.0423 (9)	0.0350 (9)	0.0264 (8)	−0.0076 (7)	−0.0101 (7)	−0.0049 (7)
C1	0.0565 (13)	0.0500 (13)	0.0232 (10)	−0.0094 (10)	−0.0137 (9)	−0.0049 (9)
C2	0.0343 (10)	0.0386 (10)	0.0235 (9)	−0.0062 (8)	−0.0066 (7)	−0.0026 (8)
C3	0.0382 (10)	0.0375 (11)	0.0265 (10)	−0.0088 (8)	−0.0083 (8)	−0.0035 (8)
C4	0.0463 (11)	0.0300 (10)	0.0238 (9)	−0.0075 (8)	−0.0123 (8)	0.0004 (7)
C5	0.0443 (11)	0.0373 (11)	0.0259 (9)	−0.0072 (8)	−0.0118 (8)	−0.0010 (8)
C6	0.0496 (12)	0.0445 (12)	0.0248 (10)	−0.0054 (9)	−0.0104 (9)	−0.0064 (8)
C7	0.0549 (13)	0.0399 (11)	0.0302 (10)	−0.0077 (9)	−0.0183 (9)	−0.0058 (8)
C8	0.0456 (11)	0.0358 (11)	0.0351 (11)	−0.0093 (9)	−0.0166 (9)	−0.0011 (8)
C9	0.0444 (11)	0.0320 (10)	0.0264 (9)	−0.0075 (8)	−0.0105 (8)	0.0006 (7)
C10	0.0625 (14)	0.0360 (12)	0.0407 (12)	−0.0123 (10)	−0.0160 (11)	−0.0031 (9)
C11	0.0419 (12)	0.0558 (14)	0.0383 (12)	−0.0108 (10)	−0.0086 (10)	−0.0008 (10)
N1A	0.098 (2)	0.0602 (15)	0.0683 (17)	−0.0126 (14)	−0.0275 (15)	−0.0228 (14)
C1A	0.0675 (16)	0.0453 (13)	0.0412 (13)	−0.0115 (11)	−0.0082 (11)	−0.0068 (11)
C2A	0.152 (4)	0.0515 (16)	0.0383 (14)	−0.0300 (19)	−0.0020 (18)	−0.0111 (12)

### Geometric parameters (Å, º)

**Table:**

S1—C2	1.692 (2)	C5—C6	1.398 (3)
O1—C9	1.371 (3)	C6—C7	1.377 (3)
O1—C11	1.429 (3)	C6—H6	0.9500
O2—C5	1.361 (3)	C7—C8	1.385 (3)
O2—H2	0.8400	C7—H7	0.9500
N1—C2	1.327 (3)	C8—C9	1.393 (3)
N1—C1	1.453 (3)	C8—H8	0.9500
N1—H1	0.8800	C10—H10A	0.9800
N2—C2	1.357 (3)	C10—H10B	0.9800
N2—N3	1.375 (2)	C10—H10C	0.9800
N2—H2A	0.8800	C11—H11A	0.9800
N3—C3	1.278 (3)	C11—H11B	0.9800
C1—H1A	0.9800	C11—H11C	0.9800
C1—H1B	0.9800	N1A—C1A	1.123 (3)
C1—H1C	0.9800	C1A—C2A	1.448 (4)
C3—C4	1.495 (3)	C2A—H2AA	0.9800
C3—C10	1.496 (3)	C2A—H2AB	0.9800
C4—C9	1.397 (3)	C2A—H2AC	0.9800
C4—C5	1.400 (3)		
			
C9—O1—C11	117.25 (18)	C5—C6—H6	120.3
C5—O2—H2	109.5	C6—C7—C8	122.0 (2)
C2—N1—C1	123.52 (19)	C6—C7—H7	119.0
C2—N1—H1	118.2	C8—C7—H7	119.0
C1—N1—H1	118.2	C7—C8—C9	118.3 (2)
C2—N2—N3	119.82 (17)	C7—C8—H8	120.9
C2—N2—H2A	120.1	C9—C8—H8	120.9
N3—N2—H2A	120.1	O1—C9—C8	124.1 (2)
C3—N3—N2	117.10 (17)	O1—C9—C4	114.70 (18)
N1—C1—H1A	109.5	C8—C9—C4	121.23 (19)
N1—C1—H1B	109.5	C3—C10—H10A	109.5
H1A—C1—H1B	109.5	C3—C10—H10B	109.5
N1—C1—H1C	109.5	H10A—C10—H10B	109.5
H1A—C1—H1C	109.5	C3—C10—H10C	109.5
H1B—C1—H1C	109.5	H10A—C10—H10C	109.5
N1—C2—N2	116.28 (19)	H10B—C10—H10C	109.5
N1—C2—S1	124.32 (16)	O1—C11—H11A	109.5
N2—C2—S1	119.40 (16)	O1—C11—H11B	109.5
N3—C3—C4	124.78 (19)	H11A—C11—H11B	109.5
N3—C3—C10	117.49 (19)	O1—C11—H11C	109.5
C4—C3—C10	117.71 (18)	H11A—C11—H11C	109.5
C9—C4—C5	119.05 (19)	H11B—C11—H11C	109.5
C9—C4—C3	120.69 (18)	N1A—C1A—C2A	178.4 (4)
C5—C4—C3	119.99 (19)	C1A—C2A—H2AA	109.5
O2—C5—C6	123.43 (19)	C1A—C2A—H2AB	109.5
O2—C5—C4	116.64 (18)	H2AA—C2A—H2AB	109.5
C6—C5—C4	119.9 (2)	C1A—C2A—H2AC	109.5
C7—C6—C5	119.5 (2)	H2AA—C2A—H2AC	109.5
C7—C6—H6	120.3	H2AB—C2A—H2AC	109.5
			
C2—N2—N3—C3	175.26 (18)	C3—C4—C5—C6	174.31 (19)
C1—N1—C2—N2	179.94 (19)	O2—C5—C6—C7	179.1 (2)
C1—N1—C2—S1	−0.2 (3)	C4—C5—C6—C7	−0.5 (3)
N3—N2—C2—N1	−1.7 (3)	C5—C6—C7—C8	0.9 (3)
N3—N2—C2—S1	178.38 (14)	C6—C7—C8—C9	−0.8 (3)
N2—N3—C3—C4	−3.1 (3)	C11—O1—C9—C8	5.1 (3)
N2—N3—C3—C10	178.83 (18)	C11—O1—C9—C4	−173.45 (18)
N3—C3—C4—C9	−77.2 (3)	C7—C8—C9—O1	−178.17 (19)
C10—C3—C4—C9	100.9 (2)	C7—C8—C9—C4	0.3 (3)
N3—C3—C4—C5	108.7 (2)	C5—C4—C9—O1	178.65 (18)
C10—C3—C4—C5	−73.2 (3)	C3—C4—C9—O1	4.5 (3)
C9—C4—C5—O2	−179.60 (18)	C5—C4—C9—C8	0.0 (3)
C3—C4—C5—O2	−5.4 (3)	C3—C4—C9—C8	−174.16 (19)
C9—C4—C5—C6	0.1 (3)		

### Hydrogen-bond geometry (Å, º)

**Table:**

*D*—H···*A*	*D*—H	H···*A*	*D*···*A*	*D*—H···*A*
O2—H2···S1^i^	0.84	2.34	3.1823 (17)	177
N1—H1···N1*A*	0.88	2.25	3.039 (3)	149

Symmetry code: (i) −*x*, −*y*+2, −*z*+2.
